# Mapping the Burden of Hypertension in South Africa: A Comparative Analysis of the National 2012 SANHANES and the 2016 Demographic and Health Survey

**DOI:** 10.3390/ijerph18105445

**Published:** 2021-05-19

**Authors:** Ngianga-Bakwin Kandala, Chibuzor Christopher Nnanatu, Natisha Dukhi, Ronel Sewpaul, Adlai Davids, Sasiragha Priscilla Reddy

**Affiliations:** 1 Division of Health Sciences, Warwick Medical School, University of Warwick, Coventry CV4 7AL, UK; 2 Division of Epidemiology and Biostatistics, School of Public Health, University of the Witwatersrand, Braamfontein, Johannesburg 2000, South Africa; 3 Department of Mathematics, Physics & Electrical Engineering (MPEE), Northumbria University, Newcastle NE 18 ST, UK; chibuzor.nnanatu@northumbria.ac.uk; 4 Department of Statistics, Nnamdi Azikiwe University, Awka PMB 5025, Nigeria; 5 Health & Wellbeing, Human and Social Capabilities (HSC) Division, Human Sciences Research Council, Private Bag X9182, Cape Town 8000, South Africa; ndukhi@hsrc.ac.za (N.D.); rsewpaul@hsrc.ac.za (R.S.); asdavids@hsrc.ac.za (A.D.); preddy@hsrc.ac.za (S.P.R.); 6 Faculty of Health Sciences, Nelson Mandela University, Port Elizabeth 6031, South Africa

**Keywords:** hypertension, Bayesian geo-additive regression, spatial modelling, South Africa, KwaZulu-Natal, Mpumalanga

## Abstract

This study investigates the provincial variation in hypertension prevalence in South Africa in 2012 and 2016, adjusting for individual level demographic, behavioural and socio-economic variables, while allowing for spatial autocorrelation and adjusting simultaneously for the hierarchical data structure and risk factors. Data were analysed from participants aged ≥15 years from the South African National Health and Nutrition Examination Survey (SANHANES) 2012 and the South African Demographic and Health Survey (DHS) 2016. Hypertension was defined as blood pressure ≥ 140/90 mmHg or self-reported health professional diagnosis or on antihypertensive medication. Bayesian geo-additive regression modelling investigated the association of various socio-economic factors on the prevalence of hypertension across South Africa’s nine provinces while controlling for the latent effects of geographical location. Hypertension prevalence was 38.4% in the SANHANES in 2012 and 48.2% in the DHS in 2016. The risk of hypertension was significantly high in KwaZulu-Natal and Mpumalanga in the 2016 DHS, despite being previously nonsignificant in the SANHANES 2012. In both survey years, hypertension was significantly higher among males, the coloured population group, urban participants and those with self-reported high blood cholesterol. The odds of hypertension increased non-linearly with age, body mass index (BMI), waist circumference. The findings can inform decision making regarding the allocation of public resources to the most affected areas of the population.

## 1. Introduction

Cardiovascular diseases (CVDs) present a major public health concern, contributing significantly to the global disease burden in both high-income countries and low-and-middle income countries (LMICs). Many sub-Saharan African (SSA) countries have experienced dramatic increases in CVD burden and its associated risk factors over a short period. Demographic and socio-economic diversities exist in the SSA region, with various populations undergoing a rapid epidemiological and demographic transition [1,2,3]. SSA countries are facing multiple burdens of diseases, with the focus of infectious and communicable diseases including HIV/AIDS, tuberculosis and malaria being shifted towards the economic and health burden associated with non-communicable diseases (NCDs), mainly CVDs [1,2]. One such modifiable risk factor of CVD is hypertension, which is a major source of premature mortality globally [4]. In South Africa, hypertension is one of the most common NCDs, responsible for premature adult mortality [5].

The South African National Health and Nutrition Examination Survey (SANHANES), conducted in 2012, found that almost a third of South Africans aged 15 or older were hypertensive [6]. The 2016 Demographic Health Survey indicated hypertension prevalence in females at 46.0% and 44.0% in males [7]. In many LMICs higher than global average prevalence has been reported, attributed to treatment non-compliance, urbanization, and the behavioural risk factors such as poor diet, physical inactivity, and alcohol and tobacco use [8]. While South Africa remains one of the most unequal societies, the country had experienced rapid urbanization and income growth resulting in lifestyle, stress and dietary changes among all South Africans. The hypertension prevalence is now relatively high and has shown increases among all population groups [6,7]. Spatial variation in hypertension and its associated risk factors was examined in a previous study [9]. However, this was based on data older than two decades. In many high-income countries, substantial heterogeneity in disease prevalence and associated risk factors was indicated when data included geographic variations [10,11]. The mapping of both hotspot (high) and cold spot (low) areas for disease prevalence can be utilized to identify hypertension clustering, its variation by population groups, as well as contextual characteristics that include ethnic or racial residential segregation, poverty, healthcare accessibility, and other socio-economic factors [12,13]. The identification of disease prevalence hotspots is pivotal in the strengthening of disease management and prevention community-based efforts, so that resource allocation and the planning, delivery and policy of health services are informed [14,15].

Therefore, the aim of this study is to investigate the geographic variation in the prevalence of hypertension in South Africa at a province level in both 2012 and in 2016, adjusting for individual level demographic, behavioural and socio-economic variables. The analyses allow for spatial autocorrelation of the data while adjusting simultaneously for the hierarchical structure and various risk factors.

## 2. Materials and Methods

### 2.1. Study Population

Data were analyzed from participants aged 15 years or older from the South African National Health and Nutrition Examination Survey (SANHANES) of 2012 and the recent South African Demographic and Health Survey (DHS) of 2016. Both are national biobehavioral surveys of the non-institutionalized population of South Africa, that assess the health status of the population using interviews, medical examinations and biomarker analysis. Both surveys employed a multi-stage disproportionate stratified cluster sampling design.

In the 2012 SANHANES, 1000 census enumeration areas (EAs) from the 2001 population census were selected from a database of 86,000 EAs. The EAs were stratified by province and locality type, while race was used as an additional stratification variable in the formal urban areas. A total of 500 EAs representative of the socio-demographic profile of South Africa were selected and 20 households were randomly selected from each EA, yielding an overall sample of 10,000 households. All individuals residing in households were eligible to participate. A total of 8166 of the 10,000 households were valid, yielding 27,580 individuals of all ages who were eligible to be interviewed, of which 25,532 (92.6%) completed the interview. Of those interviewed, 12,025 (43.6%) volunteered to undergo a medical examination of which 7148 were ≥15 years. Details of SANHANES-1 methodology are reported elsewhere [6].

The sampling frame used for the 2016 DHS was the Statistics South Africa Master Sample Frame that was created using the Census 2011 enumeration areas (EAs). EAs of manageable size were considered as primary sampling units (PSUs), whereas small neighbouring EAs were pooled together to form new PSUs. Each of the nine provinces was stratified into urban, farm, and traditional areas, yielding 26 sampling strata. Overall 750 PSUs were selected from the sampling strata and 20 residential dwelling units were selected from each PSU. All individuals residing in the dwelling units were eligible to participate. A total of 13,288 of the 15,292 selected households were occupied, of which 11,083 (83%) individuals were interviewed and 82% of females and 77% of males aged ≥15 years had their blood pressure measured.

### 2.2. Outcome Measure

The primary outcome measure was hypertension defined as blood pressure ≥ 140/90 mmHg or self-reported health professional diagnosis or on antihypertensive medication. The blood pressure classification of 140/90 mmHg is based on the 2014 South African Hypertension Practice (SAHP) guidelines [16], which are the currently applied in South African health system. Self-reported diagnosis by a health professional was measured by participants’ responses to whether a doctor, nurse, or other health professional had told them that they have high blood pressure. In both the SANHANES and DHS, three systolic and diastolic blood pressure measurements were taken from consenting individuals aged ≥ 15 years using Omron digital blood pressure monitors. Measurements were taken at intervals of 3 min or more in the DHS and after intervals of 5–10 min in the SANHANES. The mean of the second and third measures were used in the analyses.

### 2.3. Covariates

The main independent variable was the participants’ geographic province of residence. Other covariates were various individual-level variables such as socio-demographics, health behaviours, and cardiovascular comorbidities that have been known to be associated with hypertension. Sociodemographic covariates used were sex, age, ethnicity (black/African, ‘coloured’ (refers to mixed race ancestry), white and Asian/Indian), education level respondent (no education vs. primary, secondary and higher education), and wealth index (categorised into 5 quintiles). The ethnicity and racial categories used in this paper were constructed for South Africans during apartheid and prior to the current democratic era; but are used in national statistical reporting on population groups. The authors do not necessarily subscribe to its continued use. Anthropometric measures including height, weight, and waist circumference were measured. Body mass index (BMI) in kg/m^2^ was categorized as: <25: Normal, 25–29.9: Overweight, and ≥30: Obese. Health behaviours were obtained by self-report of current smoking and current alcohol use. Cardiovascular comorbidities comprised self-reported history of diabetes, high blood cholesterol, coronary heart disease (heart attack or angina), and stroke. Diabetes and high blood cholesterol were based on whether a health professional had informed the participant that they had these conditions. Environmental factors were participants’ rural or urban area of residence.

### 2.4. Statistical Analysis

**Figure 1:**
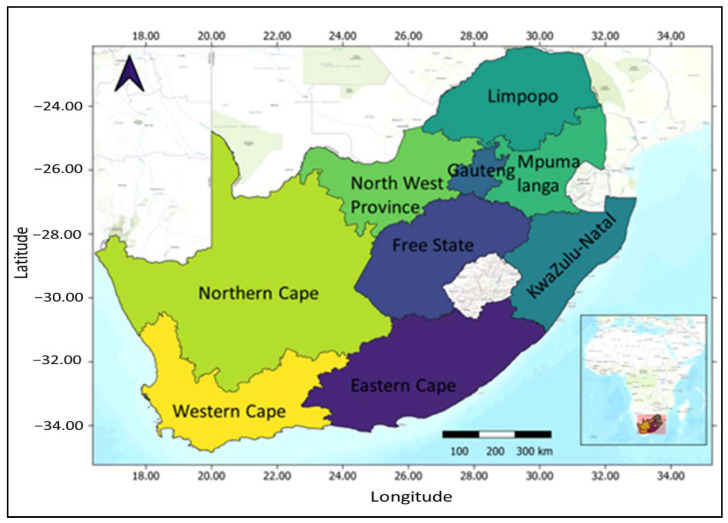
Map of South Africa showing the 9 provinces. The inset map shows the location of South Africa in the map of Africa.

Separate analyses were performed on each national survey dataset to investigate the association of various socio-economic factors on the prevalence of hypertension across the nine provinces of South Africa (Figure 1) while controlling for the latent effects of geographical location. Due to the multistage cluster sampling techniques employed in these surveys, a structured component is introduced [17] and it is no longer valid to assume that the data are independent as they are often inherently hierarchical and spatially autocorrelated. Thus, there is a need for more novel statistical analytical approaches that explicitly allow for spatially autocorrelated response while simultaneously accounting for linear and non-linear covariates and other potential sources of random errors in the data as well as data hierarchy.

Specifically, for our purposes, we utilize Bayesian geo-additive regression modelling such that the outcome variable y defined on a binary scale indicates whether an individual is hypertensive or not. Thus, y is a realization of a Bernoulli distribution with the probability mass function (PMF) $f\left(y;p\right)=p^{y}{\left(1-p\right)}^{1-y}$ for *y* ∈ {0,1}, where *p* is the probability of success, that is, the probability that a randomly selected individual is hypertensive. Then, *y* is allowed to depend on a set of key covariates through a geoadditive predictor η linked to a function of its mean with an appropriate link function h(μ) as given in Equation (1), that is,

η = Gender + Ethnicity +⋯+ angina + f(Age) + f(BMI) + f_spat (spat)(1)

where f(.) are non-parametric (smooth) functions for the non-linear effects of the continuous covariates such as Age, BMI, and Waist circumference. In addition, f_spat (.) represents the spatial random effect which account for potential spatial autocorrelation. For ease of exposition, henceforth, we shall refer to the model specified in Equation (1) as the adjusted model, such that the model is said to be unadjusted (for space or other covariates) when either f_spat () or other covariates are excluded from the model. Finally, the models are fitted in R statistical programming software version 4.0.2, (R Foundation for Statistical Computing, Vienna, Austria) via the R2BayesX, an R interface of BayesX (Georg-August-Universität Göttingen, Göttingen, Germany). All maps were produced using QGIS version 3.10. 3. (http://www.qgis.org, accessed on 17 May 2021).

## 3. Results

### 3.1. Descriptive Analyses

Characteristics of the two study populations are presented in Table 1, disaggregated by hypertensive status. The prevalence of hypertension was 38.4% in the SANHANES in 2012 and 48.2% in the DHS in 2016. In both survey years, hypertensive individuals were, on average, significantly older, more likely to be white or coloured, more likely to be living in urban areas, to be obese, and to report previous history of diabetes, high blood cholesterol, coronary heart disease and stroke (Table 1).

### 3.2. Geographic Variation in Hypertension 

**Figure 2:**
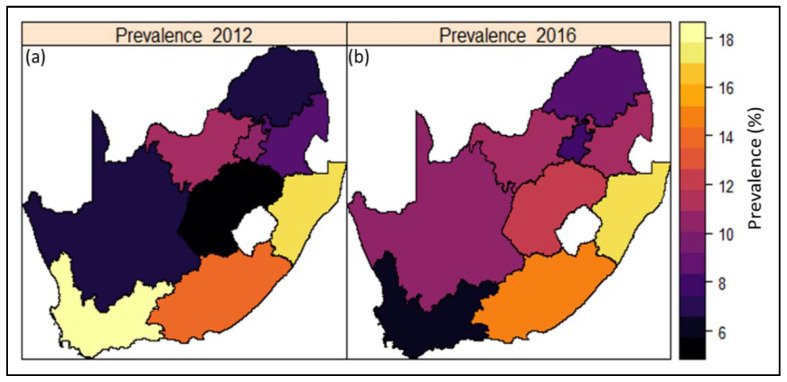
Estimates of crude prevalence of hypertension across the nine (9) provinces in South Africa based on (**a**) South African National Health and Nutrition Examination Survey (SANHANES) 2012 and (**b**) South Africa Demographic and Health Survey (DHS) 2016 datasets.

Crude estimates of prevalence of hypertension across the nine provinces in South Africa show geographical variations in prevalence for both datasets (Figure 2). In 2016 (DHS), the highest prevalence of hypertension was found in the coastal provinces of KwaZulu-Natal and the Eastern Cape, followed by Free State and Mpumalanga. Western Cape and Limpopo had the lowest prevalence in 2016 and this represents a dramatic decrease in prevalence for the Western Cape since 2012 (Figure 2b). However, prevalence remained high in KwaZulu-Natal for both surveys, whilst Limpopo remained consistently low in both surveys.

### 3.3. Bayesian Geo-Additive Regression Results

**Figure 3:**
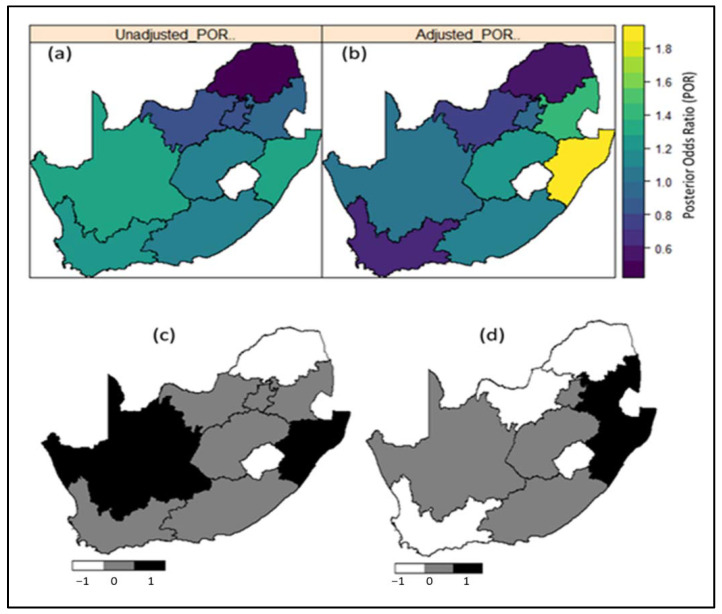
Estimates of mean posterior odds ratio (POR) of the spatial effects on prevalence of hypertension across the nine (9) provinces in South Africa based on (**a**) unadjusted model and (**b**) adjusted model; the corresponding significance maps of the posterior estimates based on 95% credible interval for (**c**) unadjusted model and (**d**) adjusted model. Evidence based on the 2016 DHS dataset. Note that in Figure 3, the central white patch (Lesotho) is excluded from the map. In Figure 3a,b, dark blue to yellow correspond to low risk to high risk provinces. In Figure 3c,d, black colour corresponds to significantly high risk regions; white colour corresponds to significantly low risk regions; and grey colour correspond to regions where the risks are not statistically significant.

**Figure 4:**
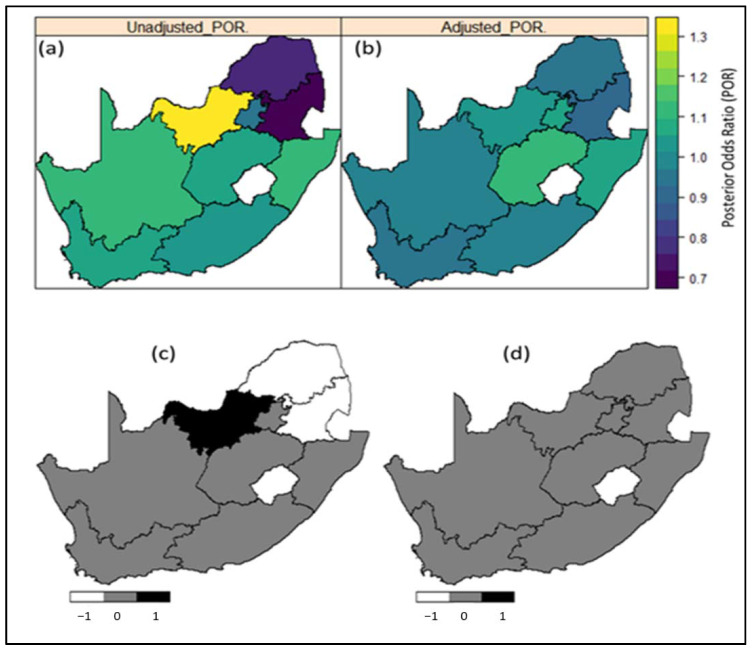
Estimates of mean posterior odds ratio (POR) of the spatial effects on prevalence of hypertension across the nine (9) provinces in South Africa based on (**a**) unadjusted model and (**b**) adjusted model; the corresponding significance maps of the posterior estimates based on 95% credible interval for (**c**) unadjusted model and (**d**) adjusted model. Evidence based on the 2012 SANHANES dataset. Blue to red correspond to low risk to high-risk provinces. Note that in Figure 3, the central white patch (Lesotho) is excluded from the map. In Figure 3a,b, dark blue to yellow correspond to low risk to high risk provinces. In Figure 3c,d, black colour corresponds to significantly high risk regions; white colour corresponds to significantly low risk regions; and grey colour correspond to regions where the risks are not statistically significant.

Table 2 presents estimates of the Posterior Odds ratio (POR) along with the corresponding measure of uncertainty-the 95% credible interval, from the various models fitted to the datasets, and the corresponding deviance information criterion (DIC) for model selection. Models with smaller DIC were retained as providing the better fit. The posterior odds ratio (POR) maps of the nine provinces in conjunction with the corresponding significance maps indicate that the risk of hypertension is significantly high in KwaZulu-Natal and Mpumalanga based on the 2016 DHS data, despite being previously nonsignificant in 2012 (Figure 3 and Figure 4).

**Figure 5:**
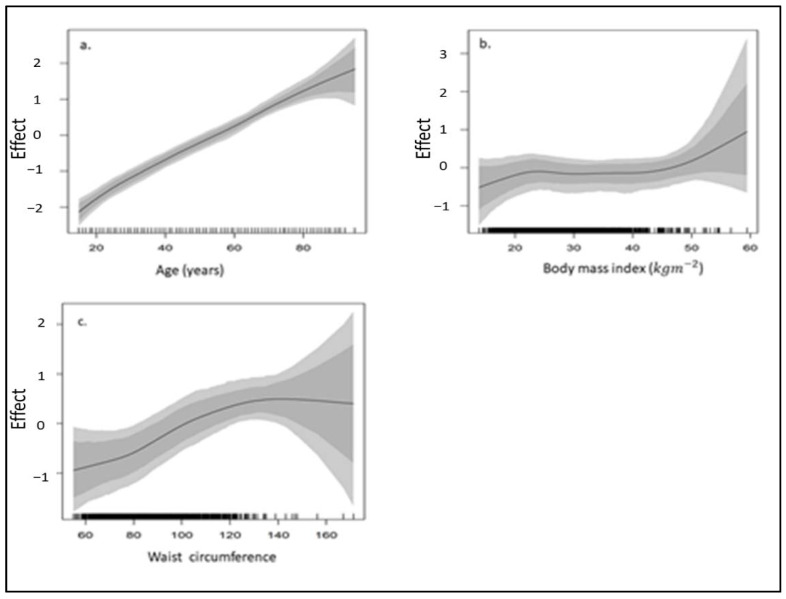
Non-linear smooth function plots of the effects of (**a**) age, (**b**) body mass index (BMI) and (**c**) waist circumference, on the prevalence of hypertension in South Africa based on the adjusted model fitted to the 2016 DHS dataset.

**Figure 6:**
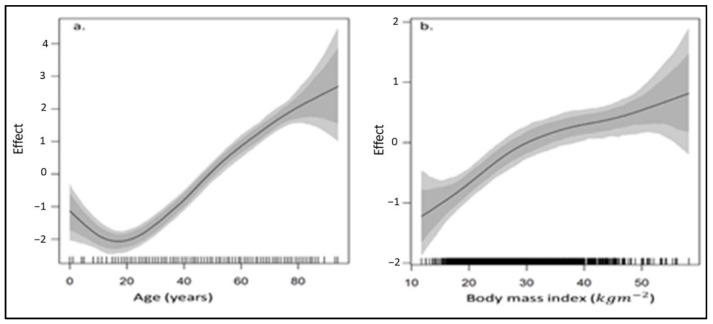
Non-linear smooth function plots of the effects of (**a**) age and (**b**) body mass index (BMI) on the prevalence of hypertension in South Africa based on the adjusted model fitted to the 2012 SANHANES dataset.

Table 2 shows that males are significantly more likely to become hypertensive than women as affirmed across the two datasets. The risk of hypertension showed positive correlation with age such that the older one gets, the higher the chances of becoming hypertensive (Figure 5 and Figure 6). Estimates based on the 2016 DHS show that South Africans who identify as coloured are about 1.67 times significantly more likely to be hypertensive than those who identify as Black/African. While the odds of being hypertensive were higher among less educated individuals, these differences were not statistically significant. People who lived in urban areas are more likely to become hypertensive than those who lived in rural areas. Additionally, the risk of hypertension increased with BMI and waist circumference (Figure 5 and Figure 6), as well as with having high blood cholesterol.

## 4. Discussion

South Africa has the largest measured hypertension prevalence in Southern Africa [5]. While many studies have focused on socio-demographic factors, geographic variation has been minimal or absent. This study therefore aimed to map the burden of hypertension in South Africa by conducting a comparative analysis of the 2012 SANHANES and 2016 DHS surveys.

It is evident from this study that hypertension is a significant public health issue in South Africa. The present study highlights both crude and adjusted estimates across the nine provinces indicating geographic variations in hypertension prevalence for both the 2012 SANHANES and 2016 DHS surveys. This study also found consistent associations with various emerging and traditional hypertension risk factors. Specifically, from the DHS dataset, age, being a drinker and smoker, excess body weight, as well as the major CVD comorbidities such as high blood sugar, high blood cholesterol, angina, and stroke were associated with a higher hypertension prevalence. From the SANHANES dataset, specifically age, excess body weight, being a current smoker, and the major CVD comorbidities were associated with a higher hypertension prevalence. This is indicative that these risk factors have continuously contributed to the exacerbation of hypertension prevalence over time. This may be due to the aging of populations and rapid urbanization, that leads to changes in their diet and lifestyle [18]. Similar patterns of association with comorbidities, smoking and alcohol drinking were observed in a previous study by Kandala et al. [9] on geographic variation in hypertension using South African data from 1998.

Estimates from both datasets indicated that South Africans who identify as coloured, those living in urban areas and have lower formal education levels were more likely to be hypertensive. This is consistent with the literature, as previous studies have indicated that having a better knowledge of hypertension was associated with improvement in compliance of antihypertensive treatment and the subsequent control of hypertension [19,20]. Rapid urbanization has resulted in populations having elevated levels in psychosocial stress which, coupled with lifestyle and dietary changes, have increased their risk of hypertension or exacerbated the disease [18].

Regarding geographic variation at a province level, after adjusting for all covariates, KwaZulu-Natal and Mpumalanga indicated significantly higher hypertension prevalence in 2016. Notably, only 55.5% of KwaZulu-Natal’s census enumeration areas were classified as urban and Mpumalanga is largely rural. While this statistical approach detects the geographic patterns of hypertension prevalence, further research is needed to explain the differences observed. One such reason could be that KwaZulu-Natal has among the highest obesity rates and obesity is a major risk factor hypertension. It could also be posited that the nutrition transitions, with their resultant changes in dietary and physical activity patterns, have progressed at a faster rate between 2012 and 2016 in these provinces than the other provinces. Notably, several districts in KwaZulu-Natal experienced very high hypertension incidence rates in 2015/2016 [21]. These findings can complement other studies on chronic disease mapping and inform public health policy and health educational programs at both national and provincial levels. The findings will also inform decision making regarding the allocation of public resources and funds to the most affected areas of the population. In addition, these provinces need to be closely monitored over time as they are identified high risk regions.

Regarding the statistical approach used in this paper, there are many potential advantages of this approach over more conventional approaches like discrete-time Cox models with time varying covariates and fixed or random districts effects; or standard 2-level multilevel modelling with unstructured spatial effects [22,23]. In the conventional models, it is assumed that the random components at the contextual level (province in our case) are mutually independent. In practice, these approaches specify correlated random residuals (see, for instance [24]), which is contrary to the assumption. Further, Borgoni and Billari [25] point out that the independence assumption has an inherent problem of inconsistency. They argue that if the location of the event matters, it makes sense to assume that areas close to each other are more similar than areas that are far apart. Moreover, treating groups (in our case districts) as independent is unrealistic and lead to poor estimates of the standard errors. As Rabe-Heskesth and Everitt [26] pointed out, standard errors for between-district factors are likely to be underestimated because we are treating observations from the same districts as independent and thus increasing the apparent sample size.

On the contrary, standard errors for within district factors are likely to be overestimated [27]. On the other hand, Demographic and Health Survey data are based on a cluster random sample of districts which, in turn, introduces a structured component. Such component allows us to borrow strength from neighbors in order to cope with the posterior uncertainty of the district effect and obtain estimates for areas that may have inadequate sample sizes or are not represented in the sample. In an attempt to highlight the advantages of our approach in a spatial context and examine the potential bias incurred when ignoring the dependence between aggregated spatial areas, we fit several models with and without the structured and random components.

The study is subject to some limitations. Firstly, the cross-sectional nature of the data limits interpretations of temporality and causality. Secondly the health behaviour and CVD comorbidity variables are self-reported, which are subject to social desirability and recall bias. Further, a known diagnosis of hypertension or other morbidities may influence self-reports of risk behaviors or vice versa. CVD comorbidity variables are self-reported because HbA1c was measured in subsamples of participants and blood cholesterol was only measured in a subsample of SANHANES, which limited the use of these measures to assess blood cholesterol and diabetes. It is often difficult to accurately measure CVD comorbidity in national population-based surveys. Thirdly, there was limited information on dietary habits and physical activity, which are important risk factors for hypertension, in one or both surveys. Fourth, blood pressure measurements were not taken at the same time of the day for all participants due to the large numbers of participants surveyed. Notably, between South Africa’s Censuses of 2001 and 2011, the country’s provincial demarcations underwent minor changes at provincial and municipal boundaries. While these changes affected small percentages of land area in some provinces, they may have minor impact on the interpretation of the results. We, therefore, suggest these limitations must be taken into account while interpreting the results of this study.

## 5. Conclusions

The provinces KwaZulu-Natal and Mpumalanga indicated significantly higher hypertension prevalence in 2016. These provinces need to be monitored over time as they are identified as high risk. In both survey years, hypertension was significantly higher among participants who were male, from the coloured population group and from urban areas. The prevalence of hypertension increased non-linearly with age, BMI, waist circumference, and blood cholesterol. The findings can and inform public health policy and health educational programs at both national and provincial levels, and inform decision making regarding the allocation of public resources to the most at-risk regions of the population, with the aim of achieving more significant reduction of the scourge of hypertension and related issues in South Africa.

## Figures and Tables

**Table 1:** Baseline characteristics of the study populations by hypertensive status.

	DHS 2016			SANHANES 2012		
	% Hypertensive: 48.2% (*n* = 8230)			% Hypertensive: 38.4% (*n* = 6867)		
Variable	Normotensive (*n* = 4227)	Hypertensive (*n* = 4003)	*p*-Value ^1^	Normotensive (n = 3827)	Hypertensive (*n* = 3040)	*p*-Value ^1^
Total	4227 (100)	4003 (100)				
Mean age (S.E)	31.6 (0.28)	47.9 (0.53)	<0.001	30.5 (0.35)	46.8 (0.73)	<0.001
Sex (%)						
Male	1719 (40.7)	1527 (37.8)		1367 (44.2)	991 (41)	
Female	2508 (59.3)	2476 (62.2)	0.04	2460 (55.8)	2048 (59)	0.128
Ethnicity (%)						
Black/African	3793 (89.9)	3335 (83)		2765 (80.8)	2005 (73.5)	
White	98 (3)	201 (6.6)		60 (5.9)	71 (11)	
Coloured	284 (5.3)	414 (8.5)		829 (9.8)	774 (12.6)	
Indian/Asian	51 (1.8)	50 (2)	<0.001	162 (3.4)	181 (3)	0.021
Education (%)						
No education	195 (4.3)	561 (12.3)		187 (4.7)	412 (9.2)	
Primary education	701 (14.6)	960 (22.7)		614 (15.3)	782 (22.4)	
Secondary education	2975 (72.1)	2123 (55.9)		2317 (71.8)	1217 (56.8)	
Higher education	324 (9)	314 (9.1)	<0.001	176 (8.3)	145 (11.6)	<0.001
Place of residence (%)						
Urban	2090 (61.1)	2229 (65)		2263 (60)	1860 (64.7)	
Rural	2137 (38.9)	1774 (35)	0.037	1564 (40)	1180 (35.3)	0.029
Wealth index						
Poorest	957 (21.2)	814 (19.8)		764 (22.1)	550 (19.6)	
Poorer	995 (21.7)	808 (18.1)		632 (20.6)	496 (17.9)	
Middle	1025 (22)	907 (21.1)		698 (22)	525 (19.5)	
Richer	760 (18.9)	858 (20.4)		677 (20.5)	569 (21.8)	
Richest	490 (16.1)	616 (20.6)	0.002	395 (14.8)	344 (21.2)	0.003
Body Mass Index (kg/m^2^) (%)						
<25 kg/m^2^	2385 (56)	1478 (35.5)		2169 (58.7)	1052 (37.6)	
25–29.9 kg/m^2^	974 (23.6)	997 (26.1)		770 (21.4)	698 (24.2)	
≥30 kg/m^2^	824 (20.4)	1457 (38.4)	<0.001	754 (19.9)	1134 (38.3)	<0.001
Waist circumference (tertile) (%)						
1 (lowest)	1840 (43.9)	841 (21.2)		1554 (42.9)	583 (20.3)	
2 (middle)	1419 (35)	1258 (31.4)		1291 (34.8)	974 (35.1)	
3 (highest)	885 (21.1)	1829 (47.3)	<0.001	835 (22.4)	1337 (44.6)	<0.001
Smoking status (%)						
Noncurrent smoker	3439 (80.9)	3208 (80.6)		2809 (83.5)	2218 (79.4)	
Current smoker	788 (19.1)	795 (19.4)	0.829	619 (16.5)	568 (20.6)	0.058
Drinking status (past 12 months) (%)						
Noncurrent drinker	340 (17)	323 (20.2)		2616 (74.1)	2157 (71.4)	
Current drinker	1394 (83)	1199 (79.8)	0.095	796 (25.9)	629 (28.6)	0.236
Diabetes (%)						
No	4152 (98.5)	3669 (91.8)		3327 (98.1)	2350 (86.9)	
Yes	60 (1.5)	314 (8.2)	<0.001	76 (1.9)	411 (13.1)	<0.001
High blood cholesterol (%)						
No	4172 (99)	3783 (93.5)		3246 (98.3)	2452 (88.5)	
Yes	36 (1)	195 (6.5)	<0.001	49 (1.7)	238 (11.5)	<0.001
Heart attack or angina (%)						
No	4134 (98)	3764 (94.6)		3333 (96.6)	2532 (92.5)	
Yes	83 (2)	227 (5.4)	<0.001	101 (3.4)	248 (7.5)	<0.001
Stroke (%)						
No	4186 (99.5)	3903 (97.6)		3398 (99.2)	2641 (95)	
Yes	29 (0.5)	92 (2.4)	<0.001	30 (0.8)	152 (5)	<0.001
Region of residence (province)						
Western Cape	204 (7.4)	244 (10.4)		621 (11.9)	549 (15.4)	
Eastern Cape	569 (12.5)	593 (14.4)		534 (12.8)	436 (12.9)	
Northern cape	323 (2)	407 (2.5)		238 (2.5)	209 (2.2)	
Free state	401 (5.2)	464 (6.3)		191 (3.5)	177 (3.5)	
Kwazulu-Natal	553 (16.1)	710 (21.9)		570 (19.9)	507 (20.8)	
Northwest	530 (9.2)	439 (6.9)		327 (5.4)	337 (6.5)	
Gauteng	391 (26)	328 (22.3)		440 (22.1)	329 (25.1)	
Mpumalanga	534 (8.5)	465 (8.3)		518 (9)	292 (6.1)	
Limpopo	722 (13.1)	353 (6.9)	<0.001	388 (12.9)	204 (7.5)	0.001

Data are expressed as weighted mean (standard deviation) or as weighted percentages with counts; ^1^ *p*-values for comparison between hypertensive and normotensive respondents. SANHANES: South African National Health and Nutrition Examination Survey; DHS: South African Demographic and Health Survey.

**Table 2:** Adjusted and unadjusted estimates of the posterior odds ratio (POR) from the Bayesian geo-additive regression models.

	DHS 2016		SANHANES 2012	
	POR		POR	
Variable	Unadjusted Mean (95% CI) DIC = 11,849.05	Adjusted Mean (95% CI) DIC = 3586.07	Unadjusted Mean (95% CI) DIC = 9248.18	Adjusted Mean (95% CI) DIC = 2580.84
**Age**		See graph (Figure 5)		See graph (Figure 6)
**Sex**				
Male	1.289 (1.071, 1.569)	1.279 (1.036, 1.563)	1.264 (1.027, 1.560)	1.317 (1.069, 1.625)
Female (ref)	1.000	1.000	1.000	1.000
**Ethnicity**				
Black/African (ref)	1.000	1.000	1.000	1.000
White	1.201 (0.789, 1.728)	1.136 (0.742, 1.716)	0.746 (0.363, 1.442)	0.683 (0.348, 1.323)
Coloured	1.703 (1.206, 2.428)	1.672 (1.216, 2.412)	1.305 (0.984, 1.748)	1.278 (0.962, 1.694)
Indian/Asian	0.630 (0.251, 1.615)	0.675 (0.263, 1.753)	0.705 (0.423, 1.174)	0.776 (0.484, 1.211)
Education				
No education	1.235 (0.797, 1.962)	1.312 (0.846, 2.022)	8.917 (0.726, 747.901)	9.241 (0.817, 328.448)
Primary education	1.198 (0.849, 1.695)	1.236 (0.879, 1.772)	0.939 (0.603, 1.408)	0.944 (0.611, 1.409)
Secondary education	1.186 (0.906, 1.582)	1.196 (0.886, 1.637)	0.998 (0.679, 1.527)	1.014 (0.671, 1.496)
Higher education (ref)	1.000	1.000	1.000	1.000
**Place of residence**				
Urban	1.217 (1.005, 1.485)	1.229 (1.016, 1.475)	0.991 (0.789, 1.239)	0.986 (0.786, 1.226)
Rural (ref)	1.000	1.000	1.000	1.000
**Body Mass Index (kg/m^2^)**		See graph Figure 5		See graph (Figure 6)
<25 kg/m^2^ (ref)	1.000			
25–29.9 kg/m^2^	1.002 (0.780, 1.268)		1.595 (1.236, 2.035)	
≥30 kg/m^2^	1.200 (0.883, 1.691)		2.067 (1.568, 2.693)	
**Waist circumference (tertile)**		See graph (Figure 5)		
1 (lowest) (ref)	1.000		1.000	
2 (middle)	1.190 (0.973, 1.466)			
3 (highest)	1.807 (1.314, 2.485)			
Smoking status				
Noncurrent smoker (ref)	1.000	1.000	1.000	1.000
Current smoker	1.022 (0.845, 1.232)	1.059 (0.887, 1.279)	1.128 (0.879, 1.447)	1.134 (0.878, 1.461)
**Drinking status (past 12 months)**				
Noncurrent drinker (ref)	1.000	1.000	1.000	1.000
Current drinker	1.148 (0.909, 1.415)	1.148 (0.936, 1.420)		
**Diabetes/High sugar**				
No (ref)	1.000	1.000	1.000	1.000
Yes	1.407 (0.827, 2.409)	1.309 (0.790, 2.287)	2.097 (1.523, 2.861)	2.066 (1.503, 2.894)
**High blood cholesterol**				
No (ref)	1.000	1.000	1.000	1.000
Yes	2.113 (1.118, 4.063)	2.017 (1.104, 4.054)	1.650 (1.331, 2.040)	1.634 (1.270, 2.025)
**Heart attack or angina**				
No (ref)	1.000	1.000	1.000	1.000
Yes	1.202 (0.761, 2.060)	1.198 (0.740, 1.899)	1.121 (0.737, 1.720)	1.226 (0.768, 1.882)
Stroke				
No (ref)	1.000	1.000	1.000	1.000
Yes	1.021 (0.467, 2.141)	1.070 (0.513, 2.242)	1.739 (0.932, 3.587)	1.744 (0.925, 3.295)
**Region of residence (province)**		See maps (Figure 3)		See maps (Figure 4)
Western cape	1.212 (0.723, 2.011)		1.086 (0.623, 2.108)	
Eastern cape	2.078 (1.473, 2.928)		1.283 (0.753, 2.373)	
Northern cape	1.942 (1.333, 2.947)		1.269 (0.689, 2.596)	
Free state	2.351 (1.591, 3.511)		1.900 (1.040, 3.944)	
KwaZulu-Natal	3.621 (2.495, 5.318)		1.547 (0.842, 3.028)	
Northwest	1.241 (0.890, 1.761)		1.547 (0.852, 3.047)	
Gauteng	1.688 (1.182, 2.445)		1.624 (0.884, 3.340)	
Mpumalanga	2.623 (1.818, 3.699)		0.826 (0.433, 1.580)	
Limpopo (Ref)	1.00		1.00	

DIC—Deviance Information Criterion; CI—credible interval; POR—posterior odds ratio; Ref—reference factor level/category.

## Data Availability

The data presented in this study are available on request from the corresponding author. The SADHS data are available on request from the DHS website https://dhsprogram.com/data/dataset/South-Africa_Standard-DHS_2016.cfm?flag=0 (accessed on 15 June 2019) and the SANHANES data are available on request from http://datacuration.hsrc.ac.za/ (accessed on 15 June 2019).
